# LDPE and PLA Active Food Packaging Incorporated with Lemon by-Products Extract: Preparation, Characterization and Effectiveness to Delay Lipid Oxidation in Almonds and Beef Meat

**DOI:** 10.3390/foods12132450

**Published:** 2023-06-22

**Authors:** Mariana A. Andrade, Cássia H. Barbosa, Sandra Mariño-Cortegoso, Letricia Barbosa-Pereira, Raquel Sendón, Giovanna G. Buonocore, Mariamelia Stanzione, Anabela Coelho, Cristina Belo Correia, Margarida Saraiva, Ana Rodríguez-Bernaldo de Quirós, Fernanda Vilarinho, Khaoula Khwaldia, Ana Sanches Silva, Fernando Ramos

**Affiliations:** 1 Department of Food and Nutrition, National Institute of Health Doutor Ricardo Jorge, Av. Padre Cruz, 1649-016 Lisbon, Portugal; mariana.andrade@insa.min-saude.pt (M.A.A.); cassia.barbosa@insa.min-saude.pt (C.H.B.); anabela.coelho@insa.min-saude.pt (A.C.); cristina.belo@insa.min-saude.pt (C.B.C.); margarida.saraiva@insa.min-saude.pt (M.S.); fernanda.vilarinho@insa.min-saude.pt (F.V.); 2 Faculty of Pharmacy, University of Coimbra, Azinhaga de Santa Comba, 3000-548 Coimbra, Portugal; framos@ff.uc.pt; 3 Associated Laboratory for Green Chemistry of the Network of Chemistry and Technology (REQUIMTE/LAQV), R. D. Manuel II, Apartado, 55142 Porto, Portugal; 4 Mechanical Engineering and Resource Sustainability Center (METRICS), Department of Chemistry, NOVA School of Science and Technology, Campus de Caparica, NOVA University Lisbon, 2829-516 Caparica, Portugal; 5 Analytical Chemistry, Nutrition and Food Science Department, Pharmacy Faculty, University of Santiago de Compostela, 15782 Santiago de Compostela, Spain; sandra.marino.cortegoso@usc.es (S.M.-C.); letricia.barbosa.pereira@usc.es (L.B.-P.); raquel.sendon@usc.es (R.S.); ana.rodriguez.bernaldo@usc.es (A.R.-B.d.Q.); 6 Instituto de Materiales (iMATUS), University of Santiago de Compostela, 15782 Santiago de Compostela, Spain; 7 Institute of Polymers, Composites and Biomaterials (IPCB-CNR), Portici, 80125 Naples, Italy; giovannagiuliana.buonocore@cnr.it (G.G.B.); mariamelia.stanzione@cnr.it (M.S.); 8 Laboratoire des Substances Naturelles, Institut National de Recherche et d’Analyse Physico-Chimique (INRAP), Pôle Technologique de Sidi Thabet, Sidi Thabet 2020, Tunisia; khaoulakhwaldia@gmail.com; 9 National Institute for Agricultural and Veterinary Research (INIAV), I.P., Rua dos Lagidos, Lugar da Madalena, Vairão, 4485-655 Vila do Conde, Portugal; 10 Center for Study in Animal Science (CECA), Instituto de Ciências, Tecnologias e Agroambiente (ICETA), University of Porto, 4200-319 Porto, Portugal

**Keywords:** industrial lemon by-products, active food packaging, antioxidant capacity, antimicrobial activity, polylactic acid, polyethylene

## Abstract

Low-density polyethylene-based packaging with 4% lemon extract (LDPE/4LE) and two polylactic-based (PLA) packaging materials with 4% and 6% lemon extract (PLA/PEG/4LE and PLA/6LE) were produced. O_2_ and water permeability tests were performed, the total and individual phenolic compounds content were measured, and the films’ antioxidant activities were determined. The films’ ability to delay lipid oxidation was tested in two model foods: almonds, packaged with LDPE/4LE, PLA/4LE and PLA/6LE for a maximum period of 60 days at 40 °C (accelerated assay); and beef meat, packaged with the PLA/6LE for a maximum period of 11 days at 4 °C. The LE improved the WVP in all of the active films by 33%, 20% and 60% for the LDPE/4LE, PLA/4LE and PLA/6LE films, respectively. At the end of 10 days, the migration of phenolic compounds through the PLA films was measured to be 142.27 and 114.9 μg/dm^2^ for the PLA/4LE and PLA/6LE films, respectively, and was significantly higher than phenolic compounds migration measured for the LDPE/4LE (15.97 μg/dm^2^). Naringenin, apigenin, ferulic acid, eriocitrin, hesperidin and 4-hydroxybenzoic acid were the main identified compounds in the PLA, but only 4-hydroxybenzoic acid, naringenin and *p*-coumaric acid were identified in the LDPE films. Regarding the films’ ability to delay lipid oxidation, LDPE/4LE presented the best results, showing a capacity to delay lipid oxidation in almonds for 30 days. When applied to raw beef meat, the PLA/6LE packaging was able to significantly inhibit lipid oxidation for 6 days, and successfully inhibited total microorganisms’ growth until the 8th day of storage.

## 1. Introduction

Extending foods’ shelf-life is one of the most challenging goals of the food industry. In order to achieve longer shelf-life periods while maintaining the quality of foods, the food industry must resort to different knowledge areas and techniques. One alternative is active food packaging.

The goal of using active food packaging is to promote positive interactions between the packaging and the packaged foods [1,2,3,4]. These interactions can be achieved by the release of bioactive substances from the packaging to the packaged food [5,6,7]. These substances’ antimicrobial and/or antioxidant properties can help to stop or inhibit microbial growth and the development of off-flavors resulting from oxidation of the food [6].

Another issue faced by the food industry is the substantial accumulation of plastic, which has become a significant challenge in the modern world. Polyethylene, which is derived from oil production, was accidently discovered in 1935 and commercialized in 1939, under the form of high-pressure polyethylene; it is now known as low density polyethylene (LDPE) [8]. In addition to being one of the cheapest synthetic polymers, it has excellent chemical resistance, and can be molded into many shapes and forms, being flexible, soft, stretchable, having good clarity, and easily being sealed with heat. These characteristics are attributed to the long chain branches present in LDPE [8,9]. Produced by the free radical polymerization of ethylene initiated by organic peroxides or other reagents, LDPE is one of the most used polymers in food packaging. According to Geyer et al. [10], from 1950 to 2015, global plastic production increased by 379 million tones. Being non-biodegradable, these polymers do not decompose, and accumulate in the environment or in landfills.

On the other hand, polylactic acid (PLA) is a biodegradable thermoplastic derived from renewable sources such as sugarcane and corn starch [9,11]. PLA has emerged as a promising alternative to the petrochemical-based polymers, being thermoplastic and highly modular [12]. Although PLA is not an optimal candidate for food packaging due to its oxygen barrier and water vapor permeability properties, these issues can be overcome with the incorporation of additives, such as plasticizers, nanoparticles, fillers and bioactive compounds such as antioxidant and antimicrobial compounds [9,11]. When compared to LDPE, PLA presents higher tensile strength, glass transition temperature and melting onset values [12]. PLA is widely used in 3D printing, as well as in medical applications due to its unique characteristics of biocompatibility, biodegradability and thermoplastic processability. PLA has even been used for the prolonged continuous release of drugs such as contraceptives, narcotic antagonists and vaccines, among others [12,13,14].

Fruit by-products originate through the manufacture of, for example, juices, pastes and jams, resulting in a considerable amount of by-products; these are often considered to be waste with low economic value [15]. Due to their chemical composition, these by-products must be disposed of in a responsible and environmentally friendly way, adding to the final product cost [16]. Fruit by-products are a significant source of antioxidant and antimicrobial compounds, namely dietary fiber and phenolic compounds [17,18]. Phenolic compounds are present in most terrestrial plants as secondary metabolites that contribute to plants’ natural defenses against UV radiation, predators and pathogenic microorganisms. They also have an important role in plants’ coloration, aroma and taste [19,20,21].

According to FAOSTAT [22], lemon (*Citrus limon*) is one of the most widely produced *Citrus* fruits in the world, with a production of 20.049.630 tons in 2020. Lemon by-products, particularly the peels, are rich in phenolic compounds, namely flavanones (eriocitrin; hesperidin; naringin; neohesperidin; neoeriocitrin; narirutin), flavones (diosmetin 6,8-di-C-β-gluc; diosmin), flavonols (rutin, quercetin, kaempferol, limocitrol) and hydroxycinnamic acids (caffeic acid, chlorogenic acid, ferulic acid, sinapic acid, *p*-coumaric acid) [23,24,25,26,27,28,29,30,31]. These peels are also rich in dietary fiber and pectin, and possess antioxidant capacities [25].

Building upon the previous study developed by Mariño-Cortegoso et al. [32], the primary objective of this research was to investigate and compare the antioxidation potentials of active LDPE and PLA packaging that were incorporated with a lemon by-product (LE) ethanolic extract, for a high-fat content food. Additionally, the antimicrobial activity of the active PLA was determined for beef meat.

## 2. Materials and Methods

### 2.1. Materials and Reagents

Methanol, absolute ethanol, petroleum ether (40–60°, ACS reagent), isooctane (spectroscopy grade Uvasol^®^), glacial acetic acid, chloroform, barium chloride dihydrate (ACS reagent, ≥99%) and iron(II) sulfate (ACS reagent, ≥99.0%) were acquired from Merck (Darmstadt, Germany). Trichloroacetic acid, thiobarbituric acid and xylenol orange sodium were acquired from Sigma-Aldrich (Madrid, Spain). Acetic acid, 6-hydroxy-2,5,7,8-tetramethylchroman-2-carboxylic acid (Trolox), 2,2-diphenyl-1-picrylhydrazyl (DPPH), Folin–Ciocalteu’s phenol reagent, sodium carbonate, and the reference phenolic standards rutin, ferulic acid, rosmarinic acid, naringenin, apigenin, caffeic acid, p-coumaric acid, gallic acid and eriocitrin were provided by Sigma-Aldrich (St. Louis, MO, USA). Hesperidin was supplied by USP (Twinbrook Pkwy, Rockville, MD, USA), and 4-hydroxybenzoic acid by Alfa Aesar (Karlsruhe, Germany). All of the standards had a purity equal to or higher than 95%. Furthermore, LDPE was purchased from Polimeri Europa, Italy, and PLA (PLA-4032D) was supplied by NatureWorksTM, Minnetonka, MN. Plasticizer poly(ethylene glycol) and PEG (Mw = 400) were purchased from Sigma-Aldrich (Madrid, Spain). Ultrapure water was obtained through an automatic system of purification (Wasselab, Navarra, Spain).

Regarding the equipment, a Heto PowerDry PL6000 freeze dryer (Thermo Fisher Scientific, Winsford, UK), an Edmund Bühler GmbH model KS-15 compact stirrer (Hechinger, Villingen-Schwenningen, Germany), a Heraeus Multifuge X3F/X3FR (Thermo Scientific, Oxford, UK), a rotary evaporator Büchi model R-210 (Labortechnik, Wettingen, Switzerland), an internal mixer (Rheomix^®^ 600 Haake, Vreden, Germany), Evolution 300 UV-Vis (ThermoScientific™, England), a Grant Instruments™ QB Series dry block heating system (Cambridge, England), a Ox-Tran (Mocon, Model 2/20, Neuwied Germany) and a PermatranW3/31 (Mocon, Bühlertal, Germany) were used.

### 2.2. By-Products’ Selection and Extraction

In a previous study by our research group, lemon by-products extract (LE) was found to present the highest DPPH free radical inhibition percentage (33.17 mg Trolox/g of extract), the highest content in total phenolic compounds (43.38 mg gallic acid equivalents/g of extract) and the highest content in total flavonoids (20.76 mg of epicatequin equivalents/g of extract) [33]. Based on these results, LE was chosen to be incorporated into active food packaging.

The lemon by-products were kindly supplied by a Portuguese juice company, Frubaça-Cooperativa de Hortofruticultores. Upon their arrival to the laboratory, the by-products were frozen and freeze-dried, grounded and homogenized. Following the extraction method described by Andrade et al. [34], the by-products were mixed with ethanol in a 1:10 ratio, agitated in a compact stirrer for 30 min and centrifuged at 10 °C at 11,952 *g*. The supernatant was transferred to an amber pear-shaped flask, and ethanol was evaporated until it was dry, at 35 °C in the rotary evaporator. The extract was removed with the help of a spatula, vacuum packaged and stored until further use, protected from the light, at −20 °C.

### 2.3. Incorporation of the Lemon Extract into LDPE and PLA

Three active films were prepared that incorporated the LE: LDPE with 4% (*w*/*w*) of LE (LDPE/4LE), PLA with 4% (*w*/*w*) of LE (PLA/PEG/4LE) and PLA with 6% (*w*/*w*) of LE (PLA/6LE).

The LE was incorporated into LDPE and PLA by direct melting, using melt mixing and hot compression. Specifically, LE was mixed with LDPE and PLA using an internal mixer (volumetric capacity of 50 cm^3^) at 50 rpm for 5 min, at 170 °C and 180 °C, respectively. In the PLA/PEG/4LE, PEG was added (15%, *w*/*w*) to prevent brittleness [35]. In the PLA/6LE, the higher amount of LE was sufficient to plasticize the polymeric matrix.

The mixtures were then pressed using a Collin P300P press at the same temperatures (*p* = 50 bars for 3 min), resulting in active films with an average thickness of approximately 100–150 microns. Pristine PLA, PLA/PEG and LDPE films were produced for comparison purposes using the same specific processing conditions.

### 2.4. Oxygen and Water Permeability Tests of Active Films

The oxygen permeabilities (Ops) of the LDPE- and PLA-based films were determined using the Ox-Tran. Samples with a surface area of 5 cm^2^ were tested at 25 °C, setting the relative humidity (RH) at the downstream and upstream sides of the film at 50%.

The water vapor permeability (WVP) was determined using infrared sensor technology with the PermatranW3/31. Samples with a surface area of 5 cm^2^ were tested at 25 °C. Permeation tests were performed by setting the relative humidity at the downstream and upstream sides of the film to 0% and 50%, respectively. A flow rate of 100 mL/min for a nitrogen stream was used. Each test was carried out in triplicate.

### 2.5. Migration Assay of Active Films

Following European Commission Regulation No. 10/2011 [36] and its amendments, migration tests were conducted on the active films (LDPE/4LE, PLA/4LE and PLA/6LE) using 95% ethanol (*v*/*v*) as a substitute for the food simulant D2 (vegetable oil) used to mimic foods with a high fat content. Samples of films were cut into specimens of 10 cm^2^ and immersed in a glass vial with 10 mL of the food simulant, the 95% ethanol (*v*/*v*). The vials were incubated in an oven for 10 days at 40 °C to simulate the storage conditions of fatty food for 30 days at room temperature. After, 9 mL of food simulant was evaporated under nitrogen flow at 40 °C, and the residue was redissolved in ultra-purified water to reach a 10-fold concentrated extract. Finally, the films extracts were filtered through 0.45 µm PTFE hydrophilic filters for further HPLC analysis. All of the determinations were performed in independent triplicates.

#### 2.5.1. HPLC–DAD Analysis

Agilent HPLC 1200 (Agilent Technologies, Inc., Waldbronn, Germany) equipment, fitted with an autosampler, a pump, a degassing system, a thermostatic column system and a diode array detector (DAD), all controlled by HP ChemStation software (version B.03.01-SR1), was used for the chromatographic analysis, following the same conditions described by Barbosa et al. [33]. The phenolic compounds were separated on a reverse-phase Kinetex EVO C18 100 Å column (150 × 3 mm internal diameter, 5 µm particle size) (Phenomenex, Torrance, CA, USA), at 30 °C. The injection volume was 20 µL, and the mobile phase flow rate was 0.6 mL/min. The following solvents constituted the mobile phase: water (solvent A) and methanol (solvent B), both acidified at 0.1% with acetic acid.

The gradient elution used was as follows: 0 min, 95% of A, 5% of B; 3 min, 90% of A, 10% of B; 10 min, 80% of A, 20% of B; 18 min, 70% of A, 30% of B; 25 min, 30% of A, 70% of B; 33 min, 0% of A, 100% of B; 33–40 min, 100% of B, and finally the gradient was returned to initial conditions with 95% of A; 41–46 min.

UV/VIS scanning was performed continuously at wavelengths between 200 nm to 400 nm. Identification of the phenolic compounds was attained by comparing the retention times and the UV/VIS spectrum obtained by the injected standards in the same conditions. Quantification of the phenolic compounds was performed at the maximum absorbance of characteristic wavelengths for the different chemical phenolic families: 278 nm for 4-hydroxybenzoic acid, 325 nm for hydroxycinnamic acids, and 360 nm for rosmarinic acid and flavonoid glycosides, except for naringenin and eriocitrin that were quantified at 300 nm using the external linear calibration curves with determination coefficients of r2 > 0.998 [32]. The HPLC analyses of the individual extract were performed in a previous study led by Barbosa et al. [33].

#### 2.5.2. Total Phenolic Content

The total phenolic content (TPC) of the food simulant after contact with the films was assessed according to the Folin–Ciocalteu colorimetric method adapted to a 96-well microplate by Barbosa-Pereira et al. [37]. The absorbance was recorded at 750 nm using a Multiskan™ FC microplate photometer (Thermo Fisher Scientific, Waltham, MA USA). All of the determinations were performed in triplicate. The quantification was carried out using a standard curve of commercial gallic acid (20–100 mg/L, R^2^ = 0.9969), and the concentration of total phenolic compounds was expressed as mg of gallic acid equivalents (GAE)/dm^2^ of film.

#### 2.5.3. Radical Scavenging Activity through DPPH Radical Assay

The antioxidant capacity of the food simulant after contact with the films was determined with the 2,2′-diphenyl-1-picrylhydrazyl (DPPH•) radical scavenging assay described by von Gadow et al. [38], adapted to a 96-well microplate by Barbosa-Pereira et al. [37]. All of the determinations were performed in triplicate, and the absorbance was measured at 520 nm using a Multiskan™ FC microplate photometer (Thermo Fisher Scientific, Waltham, MA, USA). The reduction of the radical DPPH absorbance, expressed as inhibition percentage (*IP*), was calculated using the following equation:

(1) $$IP=\frac{A0-A30}{A30}\times 100$$

where *A*0 is the absorbance at the initial time and *A*30 is the absorbance after 30 min. A linear regression curve of Trolox was used at 12.5–300 μM (r^2^ = 0.9995) to calculate the radical scavenging activity (RSA) values. The results were expressed as µmol of Trolox equivalents (TE)/dm^2^ of active film.

### 2.6. Selection of High-Fat Content Foods

To select the model foods to be packaged with the active PLA and LDPE packaging, almonds, pistachios and beef meat were chosen based on their high content in unsaturated fatty acids, which are more prone to lipid oxidation [39]. According to the United States Department of Agriculture (USDA) [40] food database, beef has a total fat content of 19.1 g/100 g, of which 8.48 g/100 g are monounsaturated and 0.51 g/100 g are polyunsaturated fatty acids. Almonds have a total fat content of 50 g/100 g, of which 32.1 g/100 g are monounsaturated and 12.5 are polyunsaturated fatty acids. Pistachios have a total fat content of 45.3 g/100 g, of which 23.3 g/100 g is monounsaturated fatty acids and 14.4 is polyunsaturated fatty acids. The three foods were packaged and stored, and their lipid oxidation statuses were measured through the thiobarbituric acid-reactive substances (TBARS) assay (see Section 2.8.1).

Beef meat was vacuum packaged and kept at 4 ± 1 °C for 6 days, with lipid oxidation measurements taken on the 0th, 3rd and 6th days. The almonds and pistachios were ground, vacuum packaged and stored at 40 °C for a maximum of 30 days. Lipid oxidation measurements were conducted on the 0th, 7th, 15th and 30th days. The performed TBARS method was described by Rosmini et al. [41], with minor changes (see Section 2.8.1).

### 2.7. Sample Preparation and Packaging

Due to its linear lipid oxidation profile, almonds and beef were chosen as the model foods to be packaged with the active packaging. Almonds in their shells were purchased in a local store in Lisbon, Portugal. The almond shells were removed using a hammer, and the almonds were soaked in hot water for a maximum of 5 min to facilitate the manual removal of the soft peel. Then, the samples were ground and vacuum packaged with the LDPE/4LE, PLA/PEG/4LE and PLA/6LE materials. The samples were kept at 40 °C for a maximum time period of 60 days, and their lipid oxidation status was assessed at the end of the 7th, 15th, 30th, 45th and 60th storage days.

The beef meat was kindly supplied by Talho Girassol, Lda, a local store in Lisbon, Portugal. The meat was separated into 35 g pieces, vacuum packaged with the active films and, unlike the almonds, kept at 4 ± 1 °C, protected from the light, for a maximum time period of 11 days. At the end of the 1st, 4th, 6th, 8th and 11th days of storage, the lipid oxidation of the beef was measured using the TBARS assay, and a microorganisms total count was performed at 30 °C.

### 2.8. Lipid Oxidation Assays

#### 2.8.1. TBARS Assay

The TBARS assay was performed in accordance with the method described by Rosmini et al. [41], with slight changes. This assay measures malonaldehyde (MDA) equivalents, MDA being one of the major products of lipid peroxidation. Briefly, 20 mL of trichloroacetic acid aqueous solution (7.5%, *w*/*v*) was added to 5 g of sample. The samples were agitated in the compact stirrer for 1 h at room temperature (23 ± 1 °C) and at 350 rpm. Then, the samples were filtered through a No. 1 Whatman paper filter, and 5 mL of the filtered solution was mixed with 5 mL of a thiobarbituric aqueous solution (2.88 mg/mL). The samples were heated to 95 °C in the heating block for 30 min, rapidly cooled in ice for 15 min, and their absorbance was measured at 530 nm against the blank assay, containing 5 mL of MilliQ™ (Sigma-Aldrich, Madrid, Spain) water instead of 5 mL of sample. 1,1,3,3-Tetramethoxypropane was used as standard, and the results were expressed in mg of malonaldehyde equivalents per kg of sample (mg MDAE/kg).

#### 2.8.2. Fat Extraction

For the *p*-anisidine and peroxide value determination assays, the fat from the almonds needed to be extracted. Briefly, 10 g of almonds were mixed with 100 mL of petroleum ether and shaken for 1 h in the compact stirrer. At the end of this period, the samples were filtered through a No. 4 Whatman paper filter, with 1 g of anhydrous sodium sulfate. Then, the petroleum ether was evaporated in the rotary evaporator at 35 °C.

#### 2.8.3. *p*-Anisidine Value Determination

The determination of the *p*-anisidine value measures the secondary compounds formed during the lipid oxidation, mainly the aldehydes [42]. For the determination of the *p*-anisidine value, the method of the Instituto Português da Qualidade [43] was applied. Briefly, 50 mg of previously extracted almond fat (see Section 2.8.2) was mixed with 12.5 mL of isooctane, and the absorbance was measured at 350 nm in a UV/VIS spectrophotometer. Then, 2.5 mL of this solution was mixed with 500 µL of *p*-anisidine diluted in glacial acid acetic (5 mg/mL), homogenized and kept in the dark for 10 min. At the end of this time period, the absorbance was measured against the blank at 350 nm. Then, the *p*-anisidine value (*AV*) was determined by Equation (2).

(2) $$AV=\frac{12.5\times (A10-A0)}{m}$$

where *A*10 stands for the absorbance of the solution at the end of the 10 min, *A*0 stands for the absorbance of the solution at 0 min, and *m* stands for the sample’s weight in g.

#### 2.8.4. Peroxide Value Determination

For the determination of the peroxide value, the method described by Shantha and Decker [44] was applied. Firstly, to prepare the iron(II) chloride solution, aqueous solutions of barium chloride dihydrate (8 mg/mL) and iron(II) sulfate (10 mg/mL) were prepared. The barium chloride solution was slowly added to the iron(II) sulfate solution under constant stirring. Then, 2 mL of hydrochloric acid (10 N) was added, and the solution was filtered through a No. 1 Whatman paper filter. This solution had to be kept in the dark.

For the determination of the peroxide value, 300 mg of almond fat, previously extracted with petroleum ether, was mixed with 9.8 mL of a chloroform and methanol solution (7:3, *v*/*v*), and the solution was briefly homogenized. Then, 50 µL of an aqueous solution of xylenol orange sodium (10 mM) was added, and the solution was briefly homogenized. Then, 50 µL of the iron(II) chloride solution was added and the solution was again briefly homogenized. The absorbance was measured at 560 nm in the UV/VIS spectrophotometer. The peroxide value was calculated through Equation (3).

(3) $$PV=\frac{As-Ab\times m}{55.84\times m0\times 2}$$

where *As* stands for the absorbance of the sample, *Ab* stands for the absorbance of the blank, *m* stands for the slope of the calibration curve using iron(III) chloride as the standard, and *m*0 stands for the mass of the sample. The number 55.84 is the atomic weight of iron (the denominator gives the concentration of Fe^2+^ oxidized to Fe^3+^ in μg) and the division by 2 was necessary to present the results in milliequivalents of peroxide per kg of sample.

### 2.9. Microbiological Analysis

Regarding the microbial analysis performed on beef meat packaged with the control and active PLA/6LE, the total microorganisms count at 30 °C was performed using the automated test TEMPO^®^ Aerobic Count-AFNOR BIO 12/35–05/13. The microbial assays were only performed in meat, since the normal degradation of almonds during shelf-life is not normally due to the action of microorganisms but to rancidity, which mainly causes organoleptic alterations.

### 2.10. Statistical Analysis

All of the experiments were conducted with three replications, and the statistical analyses of the data were performed through a one-way analysis of variance (ANOVA) and ANOVA with repeated measures, using the SPSS^®^ Statistics software version 26.0.0.0, from IBM^®^. All of the requirements necessary to carry out the ANOVA (namely, normality of data and homogeneity of variances) were validated. Any differences among the mean values were processed with Tukey’s test. Significance was defined at *p* < 0.05. The results are expressed as the means of the replicants ± standard deviation.

## 3. Results

### 3.1. Oxygen and Water Permeability Tests of Active Films

**Figure 1:**
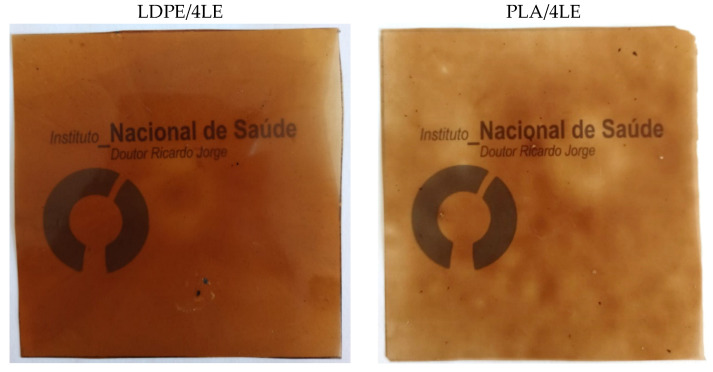
Active (LDPE/4LE and PLA/4LE) films over the Portuguese National Institute of Health logo (Instituto Nacional de Saúde Doutor Ricardo Jorge).

As can be observed in Figure 1, the LDPE/4LE and PLA/4LE active films presented a light dark-yellowish color. The films were quite homogeneous and transparent, making the Institution’s logo completely visible. The LDPE film, as expected, was the most malleable and resistant.

**Figure 2:**
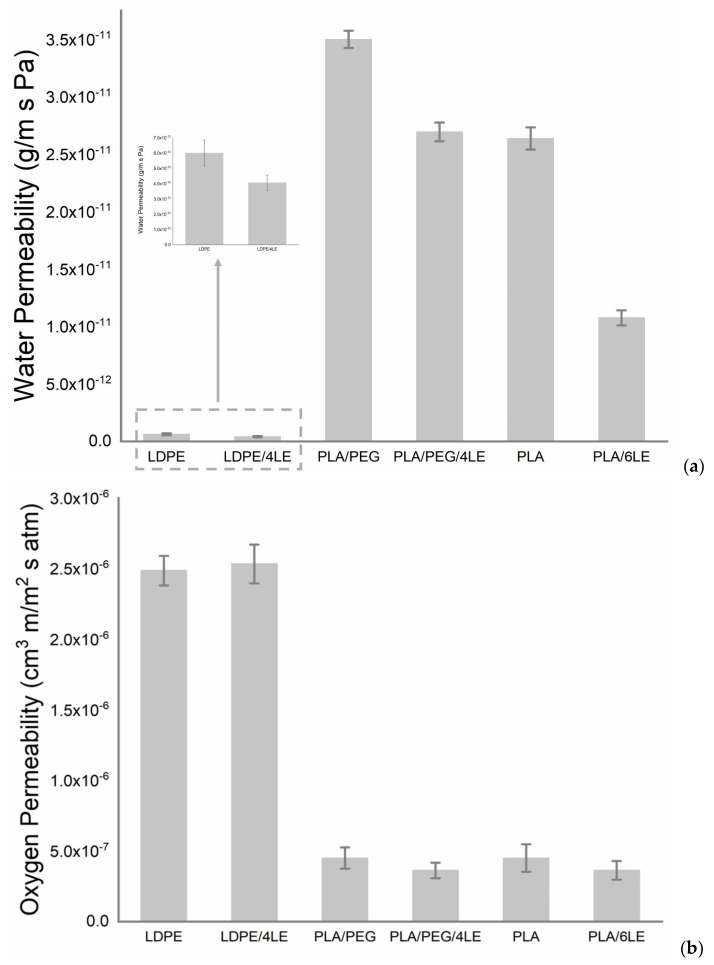
Water vapor (**a**) and O_2_ permeability (**b**) of the control (LDPE and PLA) and active (LDPE/4LE, PLA/4LE, PLA/6LE) films. Legend: LDPE—low density polyethylene; LE—lemon extract; PLA—polylactic acid; PEG—polyethylene glycol.

The WVP and OP results are exhibited in Figure 2. The presence of LE led to an improvement in the water barrier properties of the LDPE-based film, which showed a reduction of about 33% in the WVP values, which were 6.0 × 10–13 g/m s Pa and 4.0 × 10–13 g/m s Pa for LDPE and LDPE/4LE, respectively. In the case of the active PLA-based films, WVP reductions of 20% and 60% for the PLA/PEG/4LE and PLA/6LE were observed, respectively.

In the case of the OP results, the presence of the LE did not significantly modify the performance of the films: the LDPE-based films showed no reduction in OP values, whereas both PLA-based films showed a slight reduction of about 20% to pristine film. The polymer blend of the PLA/PEG behaved as a hydrophilic polymer. It had a high permeability coefficient to water due to the presence of hydrophilic groups in the PEG component of the blend. Therefore, the PLA/PEG had a higher WVP than the pure PLA film (Figure 2). It was assumed that there was a large free volume in the PLA/PE film due to the combined plasticizing effect of the PEG 400 and water. However, the addition of the LE to the LDPE, PLA or PLA/PEG decreased the free volume in the films, resulting in reductions in the WVP.

The permeability of oxygen for both the PLA/PEG/4LE and PLA/6LE was lower than the PLA/PEG and pure PLA film, respectively (Figure 2). This was due to the reduced free volume size in the PLA/PEG/4LE films in comparison with the PLA/PEG. The same pattern was observed in the PLA/6LE films in comparison with the PLA. There was a positive correlation between the OP and free volume size—a low free volume size corresponds to a low OP [35]. Between the LDPE and LDP/4LE, no differences were observed in the OP. Although the LE reduced the free volume size, the reduction was not enough to see differences in the OP.

### 3.2. Migration of Polyphenols and Antioxidant Capacity of the Active Films

The results of the migration tests performed to estimate the release of phenolic compounds from the active films to the substitute of the fat food simulant are shown in Table 1.

Based on the HPLC data, it was observed that among the active films, the PLA formulations exhibited a significantly higher release of active compounds, up to 9-fold higher than the LDPE film (*p* < 0.001). Non-significant differences were observed between the two PLA active films (*p* > 0.05), possibly due to the high standard deviation observed for the replicates of the PLA/PEG/4LE films. Despite the variability observed within the PLA/PEG/4LE replicates, the results indicated that increasing the LE content did not result in a significant increase in the overall content of active compounds released to the food simulant. This could be attributed to the possible rearrangement of the polymeric matrix, which may have affected the migration process of the active compounds.

Regarding the polyphenols determined with chromatography, the flavonoids eriocitrin, hesperidin, naringenin and apigenin were found in high concentrations in the food simulant that was in contact with PLA films. These compounds were also the most abundant in the lemon extract, as described previously by Barbosa et al. [33]. On the other hand, the LDPE/4LE films released only small amounts of three compounds. Indeed, in the LDPE films, 4-hydroxybenzoic acid was the phenolic acid that migrated in higher amounts, significantly more than for the the PLA films (*p* < 0.001).

Since it was not possible to identify and quantify all the compounds in the HPLC chromatograms, the Folin–Ciocalteu colorimetric assay was used to assess the total amount of phenolic compounds. Furthermore, for this assay, no significant differences were observed among the PLA active films. However, considering the radical scavenging activity (RSA), a slight increase was observed (*p* < 0.01), and the antioxidant capacity was higher for the PLA films where a higher concentration of lemon extract was added (PLA/6LE). This higher antioxidant capacity could be related to the migration of 4-hydroxybenzoic acid and naringenin, which were those active compounds found in significantly higher amounts (*p* < 0.001) than in the PLA/PEG/4LE films.

Considering the LDPE films, both photometric assays (DPPH and TPC) were not sensible due to the low amounts of phenolics present in the food simulant after the migration test.

### 3.3. Selection of Model Foods

**Figure 3:**
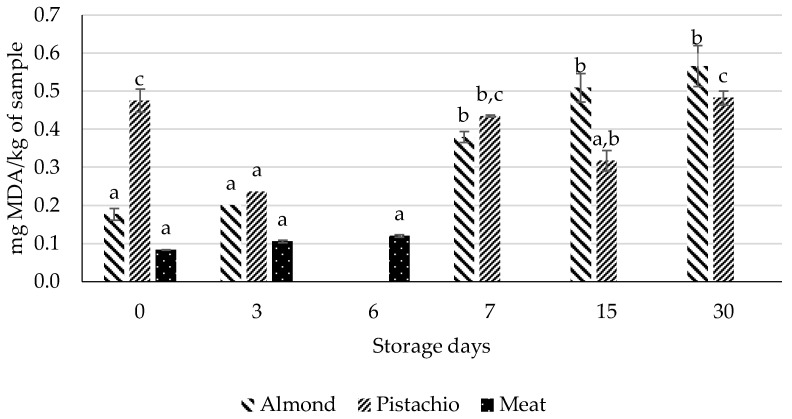
Results from the TBARS assay, evaluating the model foods beef, almonds and pistachios. Different letters represent results with significant differences. The lowercase letters compare the same sample over time.

Three foods were initially analyzed, in order to determine their suitability as model foods. Figure 3 shows that both the meat and almond samples exhibited a linear progression from the beginning of storage until the 6th (meat) and the 30th (almond) days. However, in the case of the pistachios, the lipid oxidation progression was not linear. Based on these observations, the meat and the almonds were chosen as the model foods for evaluating the effectiveness of the active packaging.

### 3.4. Lipid Oxidation Evaluation of Almond

**Figure 4:**
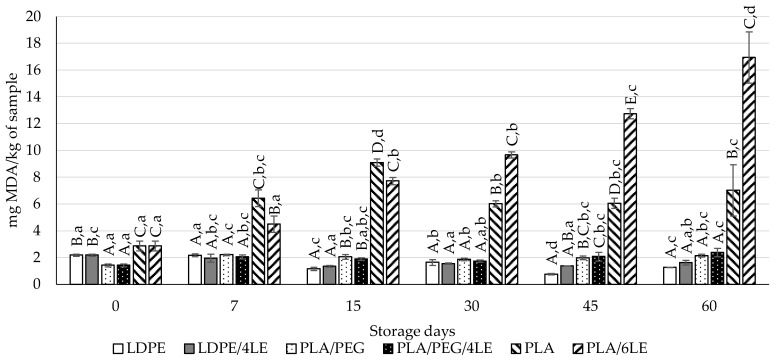
Results of the thiobarbituric reactive substances assay from almonds packed with the control (LDPE, PLA/PEG and PLA) and active (LDPE/4LE, PLA/PEG/4, PLA/6LE) films. The uppercase letters compare samples within the same storage day. The lowercase letters compare samples with the same packaging over time (for instance, LDPE at 0 and 7 days presented no significant differences, as noted by the lowercase “a”. On the other hand, on the 7th day of storage, the LDPE and LDPE/4LE presented no significant differences, as identified by the uppercase “A”). Different letters for either lowercase/uppercase comparisons represent results with significant differences.

**Figure 5:**
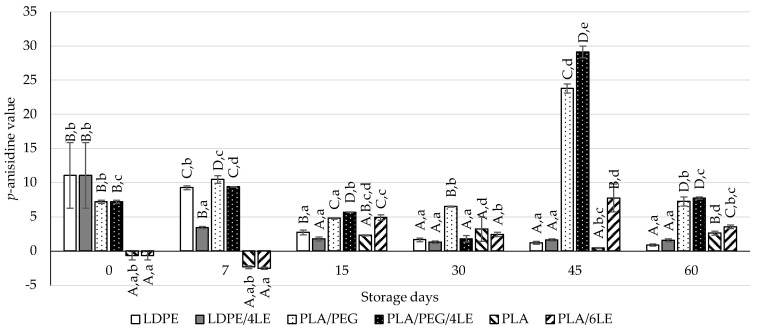
Results of the *p*-anisidine assay from almonds packed with the control (LDPE, PLA/PEG and PLA) and active (LDPE/4LE, PLA/PEG/4, PLA/6LE) films. The uppercase letters compare samples within the same storage day. The lowercase letters compare samples with the same packaging over time. Different letters for either lowercase/uppercase comparisons represent results with significant differences.

The results for the assessment of almond lipid oxidation are presented in Figure 4 and Figure 5. Both the TBARS assay and the *p*-anisidine value assay measured products resulting from the secondary lipid oxidation. The almonds that were packaged with LDPE or LDPE/4LE presented lower MDA values than almonds packaged with either PLA, with significant differences on the 15th and 45th storage days. Almonds that were packaged with the PLA and the PLA/6LE presented significantly higher MDA values throughout the storage period.

Regarding the *p*-anisidine results, on the 7th storage day, both the LDPE/4LE and PLA/PEG/4LE exhibited significantly lower *p*-anisidine values than the LDPE and PLA/PEG, respectively. The LDPE/4LE films presented significantly lower values than those of the LDPE films on the 7th and 15th storage days. Both the PLA/PEG/4LE and PLA/6LE did not present lower values than their respective controls. Both the TBARS and *p*-anisidine results indicated that the LDPE active packaging was more effective in preventing lipid oxidation of the almonds in any of the PLA packaging.

Considering the migration results (Section 3.2), the PLA/PEG/4LE and PLA/6LE films released higher amounts of active compounds that the LDPE/4LE. Combining the migration with the lipid oxidation results, it can be assumed that the active PLA packaging promoted lipid oxidation of the model food due to its significantly higher migration of active compounds compared to that of the LDPE/4LE.

The efficiency of citrus extracts was previously proven by Contini et al. [45] who demonstrated a reduction in MDA equivalents in cooked turkey meat packaged with a polyethylene terephthalate (PET) loaded with 0.46 mg/cm^2^ of a commercial citrus extract for a 4-day period. Maru et al. [46] evaluated the possible effectiveness of lemon polyphenols incorporated into chitosan and pullulan active coatings. While chitosan loaded with lemon polyphenols (3%) was able to slow the formation of MDA in poultry meat for 16 days of storage, the pullulan active coating with 3% polyphenols accelerated MDA formation at the 9th storage day [46].

**Figure 6:**
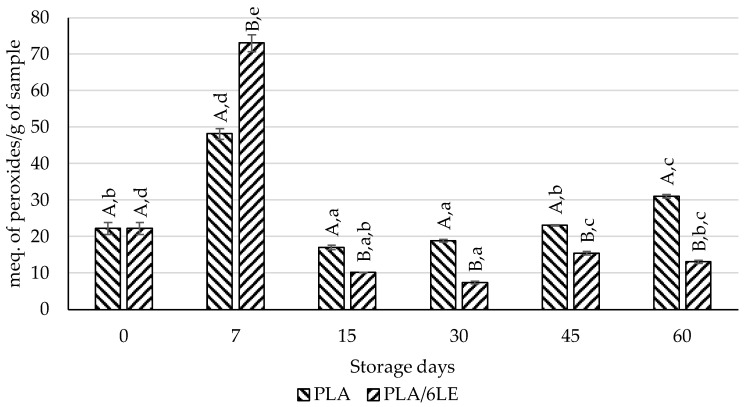
Results of the peroxide determination assay from almonds packaged with the control (PLA) and active (PLA/6LE) films. The uppercase letters compare samples within the same storage day. The lowercase letters compare samples with the same packaging over time. Different letters for either lowercase/uppercase comparisons represent results with significant differences.

Since the TBARS and *p*-anisidine results of almonds packaged with the PLA and PLA/6LE films were not clear, a peroxide determination was performed in these samples. As can be observed in Figure 6, the PLA/6LE presented significantly lower peroxide content in all of the storage days, with the exception of the 7th. The peroxide value determination is one of the most used methods for the determination of oil quality and stability [47]. This assay measures the extent of lipid peroxidation through the amount of total peroxides found in a sample, which is associated with the development of off-flavors, deterioration and toxic products in fats and oils [48].

Lipid oxidation is a complex and sequential process that is divided into three stages (initiation, propagation and termination) that occur simultaneously in a cycle until all available free radicals are consumed [47,49,50,51]. Primary lipid oxidation continually produces hydroperoxides that decompose into secondary oxidation products such as aldehydes, ketones, hydrocarbons and volatile organic acids, among others. Thus, while the TBARS and *p*-anisidine assays determine the formation of secondary oxidation products, the peroxide value assay determines the formation of primary oxidation products.

Looking at the results exhibited in Figure 5 and Figure 6, it appears that almonds packaged with the PLA and PLA/6LE films presented mostly primary oxidation products. The peak of the peroxides shown in Figure 6 for the end of the 7th day of storage suggests that the active film may have had a prooxidant effect in the matrix. This indicates that in the subsequent storage days, the formation of secondary oxidation products would increase, as can be observed in Figure 4 and Figure 5. Moreover, all of the storage assays were performed at 40 °C, in order to accelerate the foods’ oxidation and to simulate the worst storage conditions for food intended to be stored at room temperature, in accordance with European Commission Regulation no. 10/2011 and its amendments [36].

### 3.5. Lipid Oxidation and Microbial Evaluation of Beef Meat Packaged with PLA/6LE

**Figure 7:**
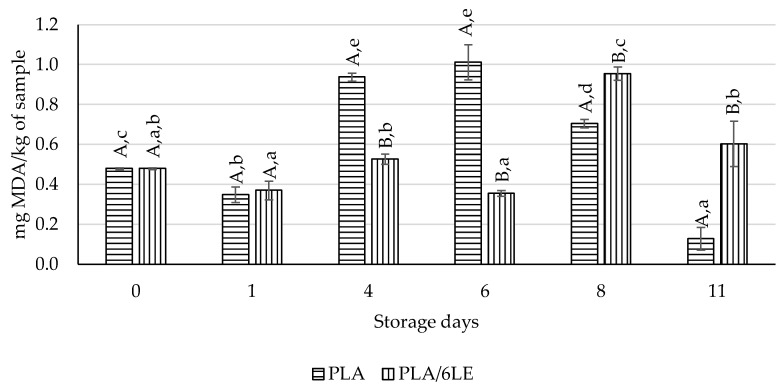
Results of the TBARS assay from meat packed with the control (PLA) and active (PLA/6LE) films. The uppercase letters compare samples within the same storage day. The lowercase letters compare samples with the same packaging over time. Different letters for either lowercase/uppercase comparisons represent results with significant differences.

**Figure 8:**
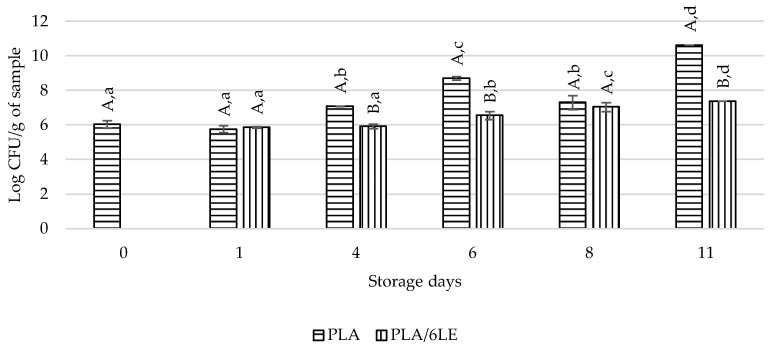
Results of the microbiological assay for meat packed with the control (PLA) and active (PLA/6LE) films. The uppercase letters compare samples within the same storage day. The lowercase letters compare samples with the same packaging over time. Different letters for either lowercase/uppercase comparisons represent results with significant differences.

The beef meat packaged with the PLA and PLA/6LE films was kept at 4 °C and analyzed at the end of 1st, 4th, 6th, 8th and 11th days of storage. The TBARS results are displayed in Figure 7, and the total microorganism counts at 30 °C are displayed in Figure 8.

The PLA/6LE films presented significantly lower MDA equivalents at the 4th and 6th storage days. The results from the 8th and 11th days also promote the results obtained for almonds packaged with the PLA and PLA/6LE films. Jiang et al. [52] described a lipid oxidation inhibition effect from grass carp collagen with chitosan encapsulated with lemon essential oil in pork meat. The authors found that the films with 30, 20 and 10% lemon essential oil presented the lowest peroxide values and malonaldehyde content. In this study, the authors also found that higher percentages of lemon essential oil in packaging showed higher peroxide values and malonaldehyde content [52].

Regarding the microbiological results, the PLA/6LE film was able to inhibit the total microorganisms count after the 1st day of storage until the 8th day of storage. On the 8th storage day, no differences were observed. The significant differences between the total microbial count were observed once more on the 11th day of storage, which shows that the antimicrobial activity of the lemon extract persisted. Furthermore, this effect was due to the good water barrier property of the active film, which hindered the entrance of water molecules into the packaging, thus creating a less humid environment to delay microbial growth. These microbiological results were in accordance with the TBARS assay results.

The antimicrobial activity of lemon by-products extracts is very well documented through the bibliography. Mexis et al. [53] demonstrated that the combined use of citrus essential oil with an oxygen scavenger was able to prolong the freshness of ground chicken meat for 2 extra days. Kadhim Hindi and Ghani Chabuk [54] showed the antimicrobial activity of aqueous extracts obtained from lemon peels against *Staphylococcus aureus*, *Enterococcus faecalis*, *Enterobacter aerogenes*, *Klebsiella pneumoniae*, *Moraxella catarrhalis* and *Pseudomonas aeruginosa*. Sanz-Puig et al. [55] also showed the antimicrobial activity of another aqueous extract obtained from lemon by-products against Escherichia coli and *Salmonella* Typhimurium. Jiang et al. [52] measured the total viable microbiological count in pork meat wrapped in grass carp collagen films with lemon essential oil encapsulated in chitosan nanoparticles; the authors found that the films with 30 and 20% lemon essential oil showed the lowest total viable microbiological counts for a storage period of 21 days, confirming the powerful antimicrobial activity of lemon by-products.

## 4. Conclusions

LE was successfully incorporated in different percentages into PLA and LDPE active packaging, and proved to be a viable additive for incorporation into new active packaging materials, from traditional non-biodegradable polymers to innovative biodegradable polymers. The migration of antioxidant compounds from the PLA/PEG/4LE and PLA/6LE films to the food simulator (ethanol 95%) was higher than the migration from the LDPE/4LE film. The LDPE/4LE material showed a higher ability to retain the LE’s phenolic compounds than the PLA films, which explains the LDPE/4LE film’s efficiency in delaying lipid oxidation in almonds stored for 30 days at 40 °C. The inability of the PLA/PEG/4LE and PLA/6LE films to retain the active LE compounds increased the amount of active compounds on the surfaces of foods since the beginning of the storage time. The higher content of phenolic compounds with high antioxidant activity promoted the lipid oxidation of the almonds.

Lemon by-product ethanolic extract has great potential to be included in the list of approved compounds/food additives, or to be applied in active packaging, similarly to rosemary extract that has been authorized by the European Union. Due to their variability among batches of natural extracts, naringenin and/or 4-hydroxybenzoic acid could be proposed as active compounds to be monitored to standardize the quality of lemon by-products extracts. Moreover, the use of by-products supports a circular economy, and fits in with the United Nations Sustainable Development Goals (UN SDGs).

## Figures and Tables

**Table 1:** The individual and total amounts of phenolic compounds that migrated from the active films (LDPE/4LE, PLA/PEG/4LE and PLA/6LE), as determined using HPLC–DAD and expressed as µg/dm^2^ of the film; Total phenolic content (TPC) expressed as µg GAE/dm^2^ film; radical scavenging activity (RSA) expressed as µmol TE/dm^2^ of the film. Results of analysis of variance (ANOVA) with Duncan’s test among different polymeric matrices developed with lemon extract at different concentrations (rows) and between the amounts of phenolic compounds within each film formulation (column).

Phenolic Compound	LDPE/4LE ^‡^ (µg/dm^2^)	PLA/PEG/4LE (µg/dm^2^)	PLA/6LE (µg/dm^2^)	Sig.
4-Hydroxybenzoic acid	9.97 ± 0.32 ^aA^	3.22 ± 0.83 ^cdC^	6.50 ± 0.40 ^eB^	***
Caffeic acid	<LOQ	3.19 ± 0.15 ^cd^	< LOQ	
*p*-coumaric acid	2.22 ± 0.95 ^c^	1.27 ± 0.20 ^d^	2.06 ± 0.11 ^f^	n.s.
Ferulic acid	<LOQ	11.4 ± 4.91 ^c^	11.0 ± 0.98 ^d^	n.s.
Eriocitrin	<LOQ	21.8 ± 3.17 ^bA^	13.0 ± 0.85 ^cB^	**
Hesperidin	<LOQ	49.9 ± 13.3 ^aA^	26.4 ± 2.1 ^aB^	*
Rutin	<LOQ	4.45 ± 0.87 ^cdA^	2.95 ± 0.12 ^fB^	*
Rosmarinic acid	<LOQ	4.06 ± 0.74 ^cd^	3.35 ± 0.11 ^f^	n.s.
Naringenin	3.77 ± 0.16 ^bC^	22.1 ± 0.42 ^bB^	27.3 ± 1.9 ^aA^	***
Apigenin	<LOQ	21.4 ± 0.50 ^bB^	22.2 ± 0.20 ^bA^	*
*Sig.*	***	***	***	
*∑* (µg/dm^2^)	15.97 ± 1.33 ^B^	142.7 ± 24.7 ^A^	114.9 ± 6.66 ^A^	***
TPC (µg GAE/dm^2^)	n.d.	624.6 ± 4.64	673.3 ± 35.9	n.s.
RSA (µmol TE/dm^2^)	n.d.	1.75 ± 0.02 ^B^	1.94 ± 0.09 ^A^	**

GAE, gallic acid equivalents; TE, Trolox equivalents; < LOQ, below limit of quantification (HPLC); n.d. = not detected. Values (mean ± standard deviation) followed by different lowercase super-indexes indicate significant differences at *p* < 0.05 among the different phenolic compounds identified in the same film, and means followed by different uppercase super-indexes indicate significant differences at *p* < 0.05 among the different polymeric matrices. Significance: * *p* < 0.05; ** *p* < 0.01; *** *p* < 0.001; n.s. = not significant. ^‡^ Data from Mariño-Cortegoso et al. [32].

## Data Availability

Data is contained within the article.
